# 166. Bring your boots: wading through GI PCR panel diagnostic stewardship applications

**DOI:** 10.1093/ofid/ofac492.244

**Published:** 2022-12-15

**Authors:** Nicholas Bennett, Sarah E Boyd, Jeffrey Sattler, Ginny Boos, Cara Johnston, Matt Humphrey, Laura Aragon, Laura Aragon, Cynthia Essmyer

**Affiliations:** Saint Luke's Health System, Kansas City, Missouri; Saint Luke's Health System, Kansas City, Missouri; Saint Luke's Health System, Kansas City, Missouri; Saint Luke's Health System, Kansas City, Missouri; Saint Luke's Health System, Kansas City, Missouri; Saint Luke's Health System, Kansas City, Missouri; Saint Luke's Health System, Kansas City, Missouri; Saint Luke's Health System, Kansas City, Missouri; Saint Luke's Health System, Kansas City, Missouri

## Abstract

**Background:**

Infectious diarrhea is a common cause of emergency department (ED) visits and hospital admissions. Polymerase chain reaction (PCR) testing allows for quick and expansive pathogen identification and facilitates earlier targeted treatment. We implemented a multiplex gastrointestinal (GI) PCR panel in 2014. In collaboration with the Antimicrobial and Diagnostic Advisement Program (ADAP), post-launch optimization strategies have changed test use. We evaluate the impact of diagnostic stewardship initiatives.

**Methods:**

GI PCR testing was initially unrestricted for ED or inpatients within 72 hours of admission. After fielding many questions regarding interpretation, the ADAP developed a guidance document in June 2019 regarding treatment considerations for all potential organisms detected. In January 2020, organism-specific treatment considerations were embedded in the test results real-time treatment guidance (figure 1). A pre-post quality improvement assessment of the changes was performed. In August 2021, individual GI PCR panel orders were replaced with an order set containing a decision tree to provide passive guidance evaluating acute vs chronic diarrhea, assessing recent antibiotic use (to consider C. difficile testing), no testing scenarios, and avoiding repeat testing (figure 2).

**Figure 1:**
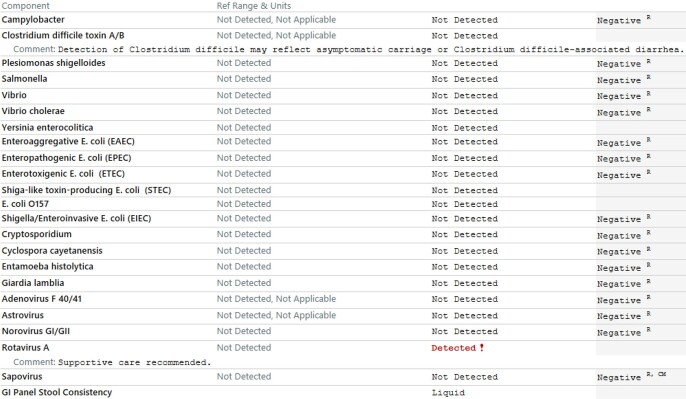
Example of templated comments for Norovirus embedded within GI PCR panel results.

**Figure 2:**
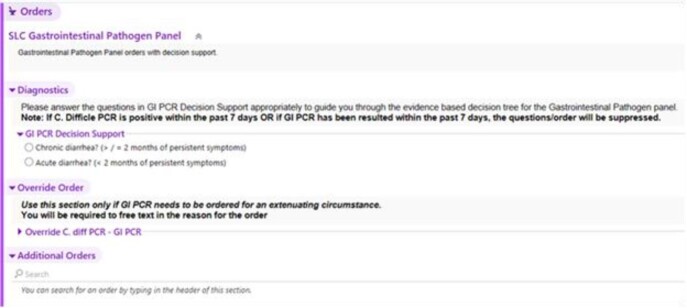
GI PCR panel order set

**Results:**

GI PCR panel use peaked in 2019 with 3,142 tests processed. The guidance document was less helpful, requiring an external site link. Embedding organism-specific GI PCR guidance significantly improved appropriate antibiotic prescribing (77.9 vs 89.1%, p=0.001). A precipitous drop off in GI PCR test orders occurred after the COVID-19 pandemic began (1,774 in 2020), partly attributed to supply chain issues. When comparing intra-pandemic years (2020 vs 2021), implementation of a smart order set was associated with a 51.3% reduction in orders (1,774 vs 864) and $131,000 in savings despite significant patient volume increases in 2021. Low use rates have persisted into the first quarter of 2022 (n=229).

**Conclusion:**

Diagnostic stewardship changes should be proactive and contextually relevant at the time of result interpretation. Antimicrobial stewardship programs are uniquely positioned to lead optimization initiatives and drive clinical and cost-effective solutions.

**Disclosures:**

**All Authors**: No reported disclosures.

